# Point of care ultrasound in pelvic health: scope of practice, education and governance for physiotherapists

**DOI:** 10.1007/s00192-022-05200-x

**Published:** 2022-05-12

**Authors:** Mike Smith, Gráinne M. Donnelly, Lucia Berry, Sue Innes, Jane Dixon

**Affiliations:** 1 College of Biomedical and Life Sciences, Cardiff University, Cardiff, UK; 2 Private Practice, Absolute Physio, Enniskillen, UK; 3 College of Health, Medicine and Life Sciences, Brunel University, London, UK; 4 School of Sport, Rehabilitation and Exercise Sciences, University of Essex, Colchester, UK; 5 Montagu House Healthcare, Alnwick, UK

**Keywords:** PoCUS, Ultrasound imaging, Scope of practice, Education, Governance, Physiotherapy, Pelvic floor, Pelvic health

## Abstract

Pelvic health and pelvic floor dysfunction have wide-reaching implications across a range of patient groups. Placing ultrasound imaging into the hands of assessing and treating clinicians (i.e. point of care ultrasound, PoCUS) can provide a step change in clinical effectiveness and efficiency. Pelvic floor dysfunction is managed by one or more members of a multi-disciplinary team that includes physiotherapists. Physiotherapists’ involvement includes diagnosis, patient education, identifying shared treatment goals, using rehabilitative strategies and empowering patients through self-management. Drawing upon existing publications in this area and applying framework principles, the authors propose a clinical and sonographic scope of practice for physiotherapists as part of supporting the consolidation and expansion of pelvic health PoCUS. Education and governance considerations are detailed to ensure the robust and safe use of this modality. Alongside empowering the use of ultrasound imaging by clinicians such as physiotherapists in the UK and internationally, we provide clarity to other members of the care pathway and ultrasound imaging professionals.

## Introduction

### Pelvic health and pelvic floor dysfunction

The term pelvic health refers to the relationship among the anatomy, physiology and functionality of the lumbopelvic region. Along with musculoskeletal elements it comprises urological, obstetric, gynaecological and colorectal conditions [1]. Pelvic floor dysfunction (PFD) may lead to various clinical presentations including urinary and faecal incontinence, emptying disorders of the bladder or bowel, pelvic organ prolapse, sexual dysfunction and pelvic pain [2]. Pelvic health issues seen by physiotherapists and the wider multidisciplinary team (MDT) include those associated with pregnancy and childbirth, the menopause and pelvic cancers. Furthermore, they can span across all life stages and apply regardless of gender.

## Care pathway components

Care pathways related to pelvic health (such as urogynaecology) typically encompass an MDT approach to management and may involve surgery, pharmacology, psychosocial and/or rehabilitation interventions [2, 3]. The selection and sequencing of these are highly individualised and will be informed by the presenting condition, clinician and patient preference, likelihood of successful outcome and resource availability.

Physiotherapists are a professional group whose input may occur at various points in a pelvic health care pathway (PHCP). Physiotherapy input may be the primary choice for patient management [4]; alternatively, it may be complementary to other interventions [2]. In this regard, physiotherapists play a key role in patient assessment and conservative rehabilitation as well as supporting the wider MDT by signposting and maximising the benefit of invasive or surgical interventions.

## Point of care ultrasound in pelvic health

Ultrasound imaging provides a dynamic, ‘bed-side’, non-ionising tool to image a wide range of tissues and organ systems, including the lumbopelvic region [5]. Whilst ultrasound imaging would historically be performed by radiologists or career sonographers within an imaging department, the application of this modality by assessing and treating clinicians (Point of care ultrasound; PoCUS) is an area of rapid expansion [6]. The emphasis here is on combining clinical assessment and ultrasound imaging to help answer a focused clinical question and/or to facilitate a particular treatment approach [7, 8]. It has the potential to play a major role in enhancing PHCPs [8]. However, the lack of a clear sonographic framework (regarding scope of practice, education and governance) within which this imaging modality is deployed raises potential concerns about safety, litigation, clarity of role and communication across the care pathway.

## Current situation for physiotherapists using PoCUS in the UK

At the time of writing there is an absence of guidance regarding the use of PoCUS (including in PHCPs) by physiotherapists in the UK. Ultrasound is a non-regulated imaging modality and thus there are no absolute barriers to its use in healthcare settings; this is compounded by the lack of protection of title for ‘sonographer’ [9]. The professional, educational and trade union body for physiotherapists in the UK (the Chartered Society of Physiotherapy, CSP) recently presented their updated ‘4 pillars of physiotherapy practice’, which includes ‘diagnostic technologies’ [10]. CSP indemnity insurance covers use of this diagnostic imaging modality where the physiotherapists’ ultrasound imaging scope of practice demonstrably aligns with their role as a physiotherapist (see section ‘Governance’ below). However, there is no published guidance on the use of ultrasound imaging by physiotherapists in the UK pertaining to what is or is not within scope of practice; this includes in PHCPs.

## Aim of this paper

The authors draw upon existing publications in this area and their collective expertise in ultrasound imaging (PoCUS and career ultrasound imaging; education and governance), PFD and the role of physiotherapists in the PHCP. In applying foundation principles (namely a framework approach to PoCUS developed by the lead author), this article presents the first attempt to identify, present and align a clinical and sonographic scope of practice for physiotherapists working in PHCPs, along with education and governance considerations. The aim is to provide guidance to pelvic health physiotherapists in the UK and their managers to incorporate ultrasound imaging into their clinical practice. For similar professional groups working in PHCPs, as well as for pelvic health physiotherapists outside of the UK, it provides a framework for integrating ultrasound imaging into their clinical practice. In parallel, it provides clarity regarding the use of ultrasound imaging to other members of the care pathway (treating clinicians such as gynaecologists, urologists, colorectal surgeons, nurses, etc.) and imaging professionals such as radiologists, sonographers and sonographer-midwives.

## Pelvic health related clinical presentations; including role of PoCUS

To contextualise the use of PoCUS by physiotherapists in PHCPs, Table 1 draws upon previous publications in the area [1, 5, 8, 11–13] to present a range of pelvic health-related clinical presentations which have been grouped together (in column 1) from a physiotherapy perspective according to commonalities of pathophysiology, anatomy or manifestation. Column 2 summarises the aims and roles of physiotherapy in each of these clinical presentations and, in so doing, provides a synopsis of the pelvic health physiotherapy specialty in the UK. Reflecting the breadth of physiotherapists’ roles and activities, these are grouped according to (1) assessment and diagnosis, (2) treatment and (3) integration with the wider MDT.

**Table 1:** Aims and role of physiotherapy for pelvic health clinical presentations, including PoCUS role

Clinical presentation	Aims and role of physiotherapy, grouped according to (1) assessment and diagnosis, (2) treatment and (3) integration with wider MDT. Potential role for ultrasound imaging in **bold**
Urinary incontinence (stress/urge/mixed/overflow*)	(1) Differentiate actual or likely cause(s) of urinary incontinence (including psychological, **anatomical/structural, muscle weakness, neurological impairment**, etc.) as a foundation for subsequent management ∆ (2) Informed by the above, treatment approaches include **education**, behavioural strategies, **pelvic floor motor re-learning, urethral support device** (3) **Communication of findings**/management approach to referring clinician and/or other care pathway members. Where appropriate, liaison with other MDT members for investigations, red flags, surgical intervention, etc.
Pelvic organ prolapse (POP)	(1) **Differentiate the presence and severity of POP ^** ∆ (2) Informed by the above, treatment approaches including **education, pelvic floor motor re-learning and strengthening**, **pessary intervention**, **voiding and defecation techniques** (3) **Communication of findings**/management approach to referring clinician and/or other care pathway members. Where appropriate, liaison with other MDT members for investigations, red flags, surgical intervention, etc.
Faecal incontinence/obstructive defecation/constipation/Obstetric Anal Sphincter Injury (OASIS)	(1) Differentiate actual or likely cause(s) of presenting symptoms (including psychological, **anatomical/structural, muscle weakness, neurological impairment**, dietary influence, side effect of medications, etc.) as a foundation for subsequent management. **Visualisation of anal sphincter complex and evaluation of motor control, recruitment quality, co-ordination and endurance of the sphincter and greater pelvic floor complex** (2) Informed by the above, treatment approaches include **education**, behavioural strategies, **pelvic floor and sphincter motor re-learning, biofeedback, defecation dynamics** (3) **Communication of findings**/management approach to referring clinician and/or other care pathway members. Where appropriate, liaison with other MDT members for investigations, red flags, surgical intervention, etc.
Pelvic pain syndromes	(1) Differentiate actual or likely cause(s) of pain (including psychological, **anatomical/structural, neurological**, etc.) as a foundation for subsequent management (2) Informed by the above, treatment approaches include **education**, behavioural strategies, **pelvic floor motor re-learning including down-training, biofeedback**, etc. (3) **Communication of findings**/management approach to referring clinician and/or other care pathway members. Where appropriate, liaison with other MDT members for investigations, red flags, surgical intervention, etc.
Recurrent urinary tract infections (RUTI)	(1) Differentiate actual or likely cause(s) of RUTIs (including **anatomical/structural, urinary retention**, dietary influence, bladder hygiene, etc., as a foundation for subsequent management (2) Informed by the above, treatment approaches include **education**, behavioural strategies, **pelvic floor motor re-learning, voiding techniques, bladder training** and hygiene, **defecation dynamics**, etc. (3) **Communication of findings**/management approach to referring clinician and/or other care pathway members. Where appropriate, liaison with other MDT members for investigations, red flags, surgical intervention, etc.
Diastasis rectus abdominis (DRA)	(1) **Differentiate the presence and severity of DRA by measuring the inter-recti distance at rest and during dynamic tests** ∆ (2) Informed by the above, treatment approaches including **education, abdominal motor re-learning and strengthening and intra-abdominal pressure strategies** ∆ (3) **Communication of findings**/management approach to referring clinician and/or other care pathway members. Where appropriate, liaison with other MDT members for investigations, red flags, surgical intervention, etc.

*Clinical reasoning should be utilised to determine the cause and potential health implications of presenting urinary retention and escalated as appropriate; e.g. suspicion of overflow incontinence secondary to a red flag such as cauda equina would require urgent referral for medical investigation

^Identification of uterus, bladder, urethra and rectum, including anatomical relationship compared to expected norm

∆Comparative evaluation in various positions, e.g. lying/standing

The use of bold text highlights where PoCUS imaging can complement or make those elements possible [5, 8]; this includes where the modality may contribute to the physiotherapist’s differential assessment, application of therapeutic intervention and patient education processes for a range of presentations.

It is noted that other conditions that relate to the pelvic region are not explicitly mentioned in column 1 yet may be of relevance. Examples such as pelvic cancers and complications of their subsequent treatment can fall within clinical presentations such as urinary incontinence and pelvic pain syndromes. Similarly, sexual dysfunction may be associated with or consequent to clinical presentations such as pelvic pain syndromes. Aligning with Table 1, Table 2 presents some key advantages conferred by the use of PoCUS by physiotherapists in PHCPs [1, 5, 8, 13–17].

**Table 2:** Advantages conferred by the use of PoCUS by physiotherapists in PHCPs

Advantages of PoCUS	Rationale	Comparator/traditional approach
Improved access to care for certain PHCP patient groups*	Non-invasive approach (i.e. PoCUS) can be well tolerated, therefore removes barrier to accessing high-quality care	Invasive procedures (e.g. digital examination or internal sEMG) which may be inappropriate or poorly tolerated
Improved clinical validity for assessment	PoCUS allows direct visualisation (and differentiation) of relevant structures (as per Table 1) PoCUS can be performed in functional positions such as standing	Digital examination of the pelvic floor or internal surface electromyography (sEMG). However, concerns regarding reproducibility with digital examination; sEMG suffers from cross-talk as confounder for differentiating individual muscle recruitment Recumbent position for digital examination
Improved patient education and understanding of their presentation (overlaps with next row)	Personalised education that is supported by real-time images and explanation (linked to the individual’s symptoms) can facilitate improved health literacy and potentially improved compliance with management	Explanation assisted by models, diagrams, leaflets, etc.
Improved clinical validity for biofeedback	Allows patient to directly visualise relevant structures during pelvic floor motor re-learning, etc. (as per Table 1)	Digital examination of the pelvic floor or internal sEMG
Enhanced treatment efficacy	PoCUS as a repeatable, objective measure to support traditional approach to clinical diagnosis. This enhances the accuracy and rationale of the clinical diagnosis reached, optimising the formulation of subsequent treatment	Clinical diagnosis based upon history taking, digital examination and sEMG
Enhanced integration with the wider MDT	Aligning PoCUS with MDT crossover of roles enables a common approach to terminology and a better approach to communicating and understanding respective roles	Largely physiotherapy-specific terminology and findings
Staff development and teaching	The visual biofeedback offered by PoCUS enables physiotherapists to develop knowledge and skills in PHCPs as part of wider understanding of the assessment and management of patients	Shadowing clinics involving traditional approaches described above in this column

*Includes paediatric, vulnerable adults (e.g. people with a learning disability), patients with pelvic pain, etc.

## A framework approach to supporting point of care ultrasound

Ultrasound imaging can be theorised to have the potential to transform the clinical effectiveness and efficiency of patient management (including by physiotherapists) in a PHCPs [18]. However, ultrasound imaging of the pelvic region can encompass a wide range of potential tissue types, organ systems, gestational processes, clinical differentials and disease processes [18]. Furthermore, there is potential for overlap with a number of other professionals who may use ultrasound imaging in this region: radiologists, career-sonographers, midwife-sonographers, obstetricians, gynaecologists, etc.

**Fig. 1.**
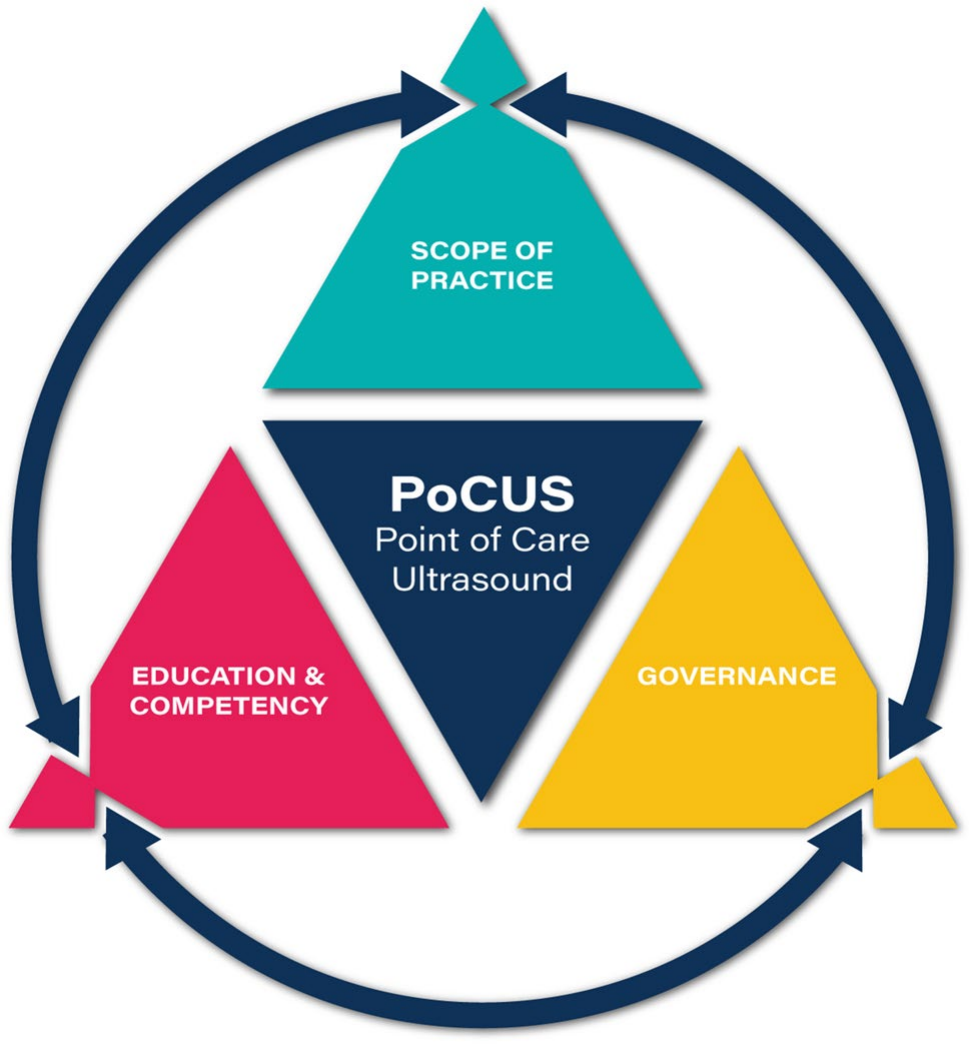
A framework for PoCUS. Concept by Dr Mike Smith (Cardiff University UK), created by Dan Molloy (freshwater.media) © Copyright 2021 Dr Mike Smith

To provide a robust foundation for the use of PoCUS by physiotherapists in PHCPs—and provide clarity to other members of the care pathway (including those who use ultrasound imaging)—we present a framework for PoCUS (Fig. 1). This comprises the elements of (1) scope of practice (ScoP), (2) education and competency and (3) governance; definitions of these terms are provided in Table 3. These terms are well established in the published literature, having been described by authors such as Lee & DeCara (2020) [19], LoPresti et al. (2019) [20], Teunissen et al. (2021) [21] and Ambasta et al. (2019) [22]. The PoCUS framework approach was devised by the lead author (stemming from longstanding work across a range of sonography and PoCUS specialities in the domains of education, work-force planning, policy and legislation) in response to a perceived need to provide comprehensive and integrated solutions for PoCUS integration into healthcare systems. The concept is that each of the elements inform and must be in alignment with each other for robust delivery of PoCUS, including for areas of activity such as PoCUS by physiotherapists in PHCPs. The application of the framework to pelvic health evolved through the collation of expert opinion via informal focus group activity and alignment with contemporary literature. At the time of writing, the PoCUS framework approach is being applied in a range of other areas of clinical practice; as such this manuscript shares some generic content with the publication Smith et al. [23].

**Table 3:** Definitions of ScoP, education and competency, and governance

Term	Key elements	Additional information
Scope of practice (ScoP)	Refers to the context and scope of the ultrasound imaging performed *plus* the interpretation/reporting of that ultrasound imaging *plus* the clinical decision making informed by that ultrasound imaging	ScoP allows for specifying any UI that is *not* going to be performed and/or where UI is performed any interpretation/reporting *not* undertaken; and/or where UI is performed any clinical decision making *not* informed by the UI
Education and competency	Refers to the education undertaken (both informally and formally) and subsequent assessments of competency	Transparent, purposeful and efficient education provision and competency assessments are made possible by aligning with the ScoP. Appropriate education and competency are key contributors to safety and governance
Governance	Includes legal and professional permissions (professional *and* regulatory body—if different), insurance arrangements and quality assurance	These are in part informed by the ScoP, and by professional and local/national agreements and via care pathway arrangements

## A framework for PoCUS by physiotherapists in PHCPs

### Scope of practice

Noting the definitions provided in Table 3, the content (in bold) in Table 1 outlines the potential breadth of the ScoP for PoCUS by physiotherapists in PHCPs. In this regard a ‘rule in’ approach is emphasised whereby clinical assessment and reasoning formulate *a priori* the likely differentials—which ultrasound imaging is then used to identify (as appropriate) [24]. This is in contrast to a ‘rule out’ approach (more typically employed by imaging services provided by imaging professionals such as radiologists and sonographers) where a range of potential sonographic findings (and subsequent clinical differentials) may be ruled out via the imaging [24].

As noted in Table 4, defining the ScoP provides clarity and thus confers a range of benefits for various key stakeholders. As part of this, clarification regarding the ultrasound imaging *not* performed and/or the interpretation/reporting *not* undertaken from that ultrasound imaging and/or the clinical decision making *not* informed by that ultrasound imaging is of equal relevance. Examples of these include:

- Gestational status or foetal imaging; this includes confirmation or exclusion of current pregnancy (including ectopic pregnancy), foetal assessment, etc.
- Prostate pathology, e.g. differentiation of benign prostatic hyperplasia from metastatic disease.
- Primary identification of fibroids, cysts or gynaecological tumours.

**Table 4:** Governance and care pathway benefits of describing scope of sonographic and clinical practice

‘Audience’	Utility of defining the ScoP (clinical and sonographic)
The referring clinician and other members of the care pathway (e.g. gynaecologist, urologist, etc.)	The referring clinician is aware of what the physiotherapist has the remit to scan and what can be inferred from the scan. Just as importantly they are aware of the limitations of the scan and that for aspects that are out of scope of practice (e.g. imaging for or identification of space occupying lesion, ectopic pregnancy, etc.) the scan is not for the purposes of either confirming or excluding
Patient	In providing informed consent, the patient is aware of what the imaging is being performed for, but just as importantly what the imaging is not being performed for (as above)
Professional body and regulatory body	The professional and regulatory bodies can identify that the imaging being performed and the clinical inferences derived from the scan are permissible for that clinician/profession and correspondingly can confer permission to proceed/professional indemnity coverage
The insurer (professional body, employer or 3rd party)	The insurer can consider the scope of sonographic and clinical practice to determine whether insurance coverage can be provided and to more accurately determine any insurance premium
The manager of the clinician	Provides clarity regarding what the clinician will be imaging and what they will be doing with that information. As such, allows for the design and staffing of existing and new care pathways
The education provider	Provides clarity regarding the requisite education content and the necessary areas for evidencing competency. This includes the clinical indication for and the clinical implementation of the sonographic information
The clinician	The clinician can undertake the necessary education and competency assessment requirements and can be confident that the relevant governance elements have been addressed and that clinicians upstream/downstream are aware of the remit of the scan

Whilst the above lie outside of a physiotherapist’s ScoP, they may be identified as either incidental or concurrent imaging findings. Just as a physiotherapist has a duty of care to escalate any suspicion of red flag signs when assessing patients in the absence of ultrasound imaging, it is also necessary that they can act upon any imaging concerns [9]. In this regard a clear protocol must be in place for the clinician to be able to discuss concerns and for the clinical assessment and/or imaging of the patient to be escalated. This could potentially include options for direct communication with those who have access to more specialist ultrasound imaging expertise, other imaging modalities and/or surgical or medical opinion. This highlights the importance of physiotherapists using PoCUS in PHCPs undertaking their ultrasound imaging as part of a wider clinical and imaging team.

### Education and competency

As per Fig. 1 and Table 3, the education and competency elements must align with and should be reflective of the ScoP. Consideration of how the clinical-physiotherapy elements of Table 1 can be learnt and competency evidenced are beyond the scope of this paper but would include both informal training and formal courses, mentoring and feedback regarding pathology, clinical reasoning and clinical management [25].

In terms of ultrasound imaging-specific education and competency, Table 5 provides a summary of key considerations regarding post-registration education and competency; this aligns with performance, interpretation and reporting on ultrasound examinations (National Occupational Standard) [26]. When combined with Table 1, these essentially provide a template for a potential ‘PoCUS by physiotherapists in PHCPs’ curriculum. Looking forwards, we advocate that educators map to these in creating the next generation of courses by which physiotherapists using PoCUS in PHCPs can robustly and comprehensively undertake their requisite learning and demonstrate initial competency.

**Table 5:** Key considerations regarding education and competency

Educational elements	Potential educational mechanisms and *assessment of competency*	Relevance to scope of practice
1. Critical understanding of how an ultrasound image is generated. Includes: • Fundamental physics as applied to ultrasound imaging • Artefacts and how to manage/interpret	Face-to-face teaching and/or provision of online/pre-reading material *Multiple choice questions/coursework around imaging scenario(s)*	As core underpinning principles, PoCUS users require an awareness of the limitations of the modality and how to interpret the sonographic representation of tissues
2. Image optimisation. Includes: • The function of ultrasound machine settings (relating back to fundamental physics principles) • ‘Knobology’ and application of image optimisation strategies in practical scenarios	Include provision of online/pre-reading material. However hands-on teaching is essential—for example using phantoms, simulators, healthy subjects *Overlap with 1. However hands-on assessment is essential and could be integrated with objective structured clinical examination (OSCE) format*	Image optimisation techniques are essential for high-quality imaging practice and allow for adaptation to different ultrasound machines and clinical scenarios
3. Safety and professional considerations. Includes: • Thermal and non-thermal effects; ALARA (As Low As Reasonably Achievable) principles • Infection prevention and control • Use of evidence-based protocols taking and labelling of standardised views • Reporting terminology • Secure storage of images and their integration into the electronic patient record of the wider care pathway • Awareness of benefits and limitations of ultrasound imaging and awareness of role of other imaging modalities • Indications for performing a scan; includes informed patient consent	Include provision of online/pre-reading material, although practical teaching is essential *Overlap with 1 and 2. Hands-on assessment is essential and could be integrated with OSCE format*	Safety considerations that are generic in ultrasound imaging and specific to pelvic region scanning Standardised image taking, recording and reporting allow for consistency with other ultrasound imagers As professionals without a pre-existing foundation in imaging, awareness of the indications for, and the role of, other imaging modalities is essential
4. Imaging of ‘normal’ anatomy. Includes: • Ability to use standardised protocols, recognise normal anatomical variation and adapt imaging based upon factors such as high levels of adipose tissue, poor patient positioning or poorly imaging tissues	Include provision of online/pre-reading material. However hands-on teaching is essential—using simulators and more importantly healthy subjects. Requires a range of ‘normal’ presentations. *Overlap with 1 and 2. However hands-on assessment is essential and could be integrated with OSCE format*	Awareness of the range of ‘normal’ presentations provides a reference for identifying deviations from normal Provides an opportunity to familiarise self with strategies for addressing sub-optimal imaging prior to moving onto imaging ‘non-normal’
5. Imaging of ‘non-normal’ anatomy. Includes: • Awareness of the range of sonographic presentations associated with different pathologies/clinical scenarios. Where applicable, how to perform a differential sonographic diagnosis • How to adapt imaging based upon factors such as high BMI, poor patient positioning or poorly imaging tissues • Clinical relevance (or otherwise) of sonographic findings, including false positive/negaitve	Include provision of online/pre-reading material. However hands-on teaching is essential—using simulators and more importantly patients. Requires a range of different pathologies/clinical presentations Essential requirements include availability of suitably qualified and experienced mentor, access to an appropriate patient mix and directly supervised scanning *Overlap with 1 and 2. However hands-on assessment is essential. Directly supervised assessment of scanning patients is the recommended assessment approach along with logbook of scans undertaken and critical reflection upon subsequent clinical decision making*	Awareness of the range of pathological/clinical presentations, including spectrum of severity. Ability to adapt imaging practice to address sub-optimal imaging An awareness of how to interpret the imaging findings, implement them into clinical decision making/treatment—and communicate them to the other care pathway members (as appropriate)

In the same manner, if an individual were to undertake a pre-existing course (e.g. via ISUOG, short courses by recognised experienced clinicians skilled in pelvic health ultrasound imaging, etc.) then mapping across to the content in Tables 1 and 5 would provide a foundation for determining whether the requisite education and competency components are addressed.

Regardless of the course type, key considerations for course providers, individual learners and their managers include: whether the full range of foundation and speciality-specific elements are taught and assessed, whether the course has been externally scrutinised by a body such as the Consortium for the Accreditation of Sonographic Education (CASE) and the importance of demonstrable competency via a formal assessment route in terms of any subsequent need to defend the clinical practice of an individual [27]. As emphasised in Table 5, availability of suitably qualified and experienced mentor(s) and access to an appropriate patient mix for directly supervised scanning are crucial components of PoCUS training. However, they are also widely acknowledged as bottlenecks in PoCUS training [28] and this is likely to be particularly acute where a specialism is developing PoCUS capacity. Mechanisms to potentially address this include accessing mentorship and observed practice at another unit or Trust—or via other professionals such as midwife-sonographers who may have overlapping areas of PoCUS practice. Key considerations here include the time burden involved with observing and being observed; honorary contracts and reciprocal working arrangements may need to be considered.

It is acknowledged that courses that meet the above considerations and that are specifically tailored to physiotherapists using PoCUS in PHCPs are not—at the time of writing—available in the UK. In the immediate and short term a pragmatic approach could include:

- For an individual physiotherapist or a service that currently provides PoCUS in PHCP:
  - Consider whether they have undertaken the relevant *foundation* educational elements (column 1 of rows 1–3 in Table 5)
    Identify the relevant elements(s) of ScoP (in Table 1) that they currently provide and consider whether they have undertaken the relevant educational elements (column 1 of rows 4 and 5 in Table 5).
  - Where the above identify any shortcomings, then the individual physiotherapist or service should consider either (1) undertaking learning in the requisite areas (this could be self-directed, short-course provision or existing courses in PHCP ultrasound imaging by other professionals such as midwives, obstetric/gynaecology sonographers, etc.) or (2) amending their current ScoP to align with those areas where there are not shortcomings.
  - For aspects where no formal assessment of competency has previously been undertaken, consider options such as (1) undertaking and documenting formal reviews of technique, image generation and interpretation with a suitably experienced professional; (2) embedding ongoing quality assurance mechanisms such as audit, double-scanning lists, etc.
- For an individual physiotherapist or a service that is looking to provide PoCUS in PHCP:
  - Using a combination of the educational elements in column 1 of rows 1–5 (Table 5) and the relevant elements(s) of ScoP (in Table 1) that they intend to provide….
  - ….Consider current educational opportunities (e.g. short-course provision or existing courses in PHCP ultrasound imaging by other professionals) and map these against the educational elements and ScoP identified in the sentence above.
  - The above should allow for pragmatic addressing of educational requirements. Where possible these should include formal assessments of competency. If not possible, consider the mechanisms mentioned above.

Due to the necessity for high-level clinical reasoning skills (required to appropriately choose to use ultrasound imaging and to integrate those findings into patient management), a physiotherapist using PoCUS in a PHCP requires a substantial level of PHCP clinical skills and experience. As such, training in PoCUS should occur at post-graduate level and by someone with the appropriate level of experience in PHCP care which is relevant to their subsequent PoCUS in PHCP ScoP.

### Governance

The use of a medical imaging modality by clinicians without a background in career imaging inevitably raises governance questions. For physiotherapists in the UK who are members of the CSP (with the relevant level of CSP provided indemnity insurance) then the key consideration is that the scope of practice must demonstrably align with their role as a physiotherapist [29]. In addition they must be able to evidence that they are appropriately trained and have been deemed competent to perform that activity. As such, the ScoP outlined in Table 1 aligns with the role of a physiotherapist working in PHCP. Combined with the above education and competency considerations, these provide the foundation for physiotherapists to use PoCUS in PHCPs in a robust manner.

It should be noted that if a physiotherapist were to use ultrasound imaging in a manner that is demonstrably outside of ScoP, e.g. akin to that of a career-sonographer or midwife-sonographer, then the above would not apply. Instead they would need to ensure vicarious liability coverage via their employer or private insurance coverage if working privately. Caveats around appropriate training and demonstrable competency for such roles would apply.

As noted in Table 4, clarity regarding the current ScoP for a physiotherapist using PoCUS in PHCP facilitates awareness by other care pathway members of what the scan is and is not undertaken for and also supports clinical managers in care pathway design and staffing. The use of terminology to explicitly clarify the nature of the scan is encouraged. An example of the professional context to the imaging process that could be communicated to colleagues is: “Aligning with the scope of clinical and sonographic practice outlined for physiotherapists working in pelvic health care pathways using point of care ultrasound in the UK [30], this ultrasound scan is undertaken for the purposes of assessing pelvic floor function and pelvic organ support as an adjunct to pelvic physiotherapy management. The identification of other anatomical or pathological elements is explicitly beyond the scope of practice of the clinician. Therefore, the scan cannot be relied upon to either confirm or exclude any such anatomical, gestational or pathological elements.”

Quality assurance considerations include data protection, storage of images, continuous professional development and access to a second opinion [6]. As PoCUS is often undertaken in non-radiology settings, direct access to PACS (picture archiving and communication system) for secure storage and backing up of sonographic images may not be available. This poses a risk to data security as well as continuity of care and the ability to review image quality. Mechanisms for the secure storage of sonographic images will need to be addressed and this may include bespoke mechanisms to upload to PACS, use of secure cloud storage (or the use of other secure image storage capacity as advised by a data compliance officer) and reporting systems which can integrate with pre-existing patient record systems.

As part of best practice, physiotherapists using PoCUS in PHCPs should undertake ongoing audit of their practice. Double-scanning with an experienced colleague and discussion of complex cases with a more experienced imager should also be undertaken as part of continuing professional development and quality assurance activities [31].

## Broader considerations

### Expansion of scope of practice

The describing of a clinical and sonographic scope of practice is not intended to stifle innovation or development of physiotherapy clinical practice or roles in PHCPs. The inclusion of both observational/feedback and differential diagnostic roles [5] within the ScoP presented in Table 1 is intentional. It reflects both the high-level skills and autonomy of physiotherapists using PoCUS in PHCPs and the transformative impact of equipping physiotherapists in PHCPs with this imaging modality.

In terms of future expansion of ScoP, this could include aspects such as the evaluation of levator ani avulsion injury, endoanal ultrasound imaging and intravaginal scanning [13, 32] and similarly the use of less commonly used ultrasound imaging techniques such as sheer wave elastography [33]. Applying the principles outlined in this paper means that where the activity demonstrably sits within the physiotherapy management of a patient, then professional regulation and CSP insurance considerations would conceivably have already been addressed. Following this, education and demonstrable competency considerations would need to be satisfied along with agreement with clinical managers—at which point such a role could be undertaken.

### Research

Evidence relating to the ability of ultrasound imaging to identify different tissues and disease processes can be drawn from a range of PoCUS and traditional ultrasound imaging (e.g. radiology and career sonography) sources. Nonetheless it is essential to add to the evidence base relating to if, where and how PoCUS can enhance clinical effectiveness and efficiency of healthcare components and pathways. This includes consideration of optimal education and service delivery models as well as whether the use of imaging may have a negative impact on clinical outcomes or efficiency of resource use. The patient experience of being scanned by a physiotherapist (and as part of an assessment/treatment episode) is also an important area to research.

In relation to physiotherapists using PoCUS in PHCPs then evidence can be drawn from other professional groups such as gynaecologists, urologists and sports medicine. Nonetheless the specifics of how physiotherapists in PHCPs work needs to be reflected. The degree of overlap for aspects of clinical practice for advanced pelvic health practitioners and other PHCP MDT members, e.g. continence nurses, gynaecologists, urologists, etc., provides a potential opportunity for pooled research and inter-professional collaboration.

### Alignment with the advanced practice agenda

As a progressive area of highly skilled practice, physiotherapists using PoCUS in PHCPs would seem to naturally align with the advanced practice agenda [34]. We advocate though that PoCUS in PHCP has the potential to become a routine part of PHCP physiotherapy practice and that as such these clinicians do not *need* to be operating at ‘advanced level’ or above. Nonetheless, the four pillars of advanced practice (clinical practice, leadership and management, education and research) overlap substantially with the expanding role that is the use of PoCUS in PHCPs by physiotherapists. As such we encourage PoCUS in PHCP adopters to explore how use of the imaging modality can further advance clinical practice and consultant roles.

### A way forward for the use of PoCUS by Physiotherapists in PHCPs

This article presents the authors’ proposals for the use of PoCUS by physiotherapists in PHCPs. To further progress this area of clinical and sonographic practice, the opinion of national [e.g. the ‘Pelvic, Obstetric and Gynaecological Physiotherapy’ (POGP) professional network of the CSP] and international societies [e.g. the International Urogynecological Association (IUGA)] should be sought. This should include where further refinements, amendments, omissions or additions to the ScoP (Table 1) are warranted, including where different professional or regulatory restrictions in other settings may apply. This process should be undertaken in parallel to consultation with government and/or licensing bodies so that governance implications (see Fig. 1 and Table 3) are aligned with and/or can be updated. In the same way, it is essential to ensure that guidelines and processes fit with individual organisations’ local policies regarding aspects such as infection control, documentation, etc.

### A direction of travel for other specialities and geographical regions

This article specifically reflects the situation for physiotherapists in the UK and, in this regard, it is noted that the level of autonomy enjoyed is greater than that of some professionals and also physiotherapists/‘physical therapists’ in some other countries. It is hoped therefore that the generic mechanisms outlined in this article will provide a potential roadmap for such professions and regions to advance their use of ultrasound imaging in a robust and sustainable manner.

## Conclusion

Reflecting the potentially transformative role of PoCUS in PCHPs, this article has presented a combined clinical and sonographic ScoP for physiotherapists in the UK. Using a framework approach, this has been informed by and aligned with education & competency and governance considerations to provide a robust foundation for PoCUS to support the clinical management of patients with urogynaecological and pelvic floor dysfunction. The mechanisms outlined are applicable to other members of the PCHP as well as physiotherapists outside of the UK.

## Abbreviations

PoCUS: Point of care ultrasound
PFD: Pelvic floor dysfunction
PHCP: Pelvic health care pathway
UK: United Kingdom
ScoP: Scope of practice
CASE: Consortium for the Accreditation of Sonographic Education
CSP: Chartered Society of Physiotherapy
PACS: Picture archiving and communication system
